# Bioinformatics Analysis Reveals the Potential Diagnostic Biomarkers for Abdominal Aortic Aneurysm

**DOI:** 10.3389/fcvm.2021.656263

**Published:** 2021-07-20

**Authors:** Xinsheng Xie, En ci Wang, Dandan Xu, Xiaolong Shu, Yu fei Zhao, Daqiao Guo, Weiguo Fu, Lixin Wang

**Affiliations:** ^1^Department of Vascular Surgery, Xiamen Branch, Zhongshan Hospital, Fudan University, Xiamen, China; ^2^Department of Vascular Surgery, Zhongshan Hospital, Fudan University, Shanghai, China; ^3^Vascular Surgery Institute of Fudan University, Fudan University, Shanghai, China; ^4^Department of Neurology, Quanzhou First Hospital Affiliated to Fujian Medical University, Quanzhou, China

**Keywords:** signaling pathways, diagnostic biomarkers, differentially expressed genes, bioinformatics analysis, abdominal aortic aneurysm

## Abstract

**Objectives:** Abdominal aortic aneurysms (AAAs) are associated with high mortality rates. The genes and pathways linked with AAA remain poorly understood. This study aimed to identify key differentially expressed genes (DEGs) linked to the progression of AAA using bioinformatics analysis.

**Methods:** Gene expression profiles of the GSE47472 and GSE57691 datasets were acquired from the Gene Expression Omnibus (GEO) database. These datasets were merged and normalized using the “sva” R package, and DEGs were identified using the limma package in R. The functions of these DEGs were assessed using Cytoscape software. We analyzed the DEGs using Gene Ontology and Kyoto Encyclopedia of Genes and Genomes pathway enrichment analysis. Protein–protein interaction networks were assembled using Cytoscape, and crucial genes were identified using the Cytoscape plugin, molecular complex detection. Data from GSE15729 and GSE24342 were also extracted to verify our findings.

**Results:** We found that 120 genes were differentially expressed in AAA. Genes associated with inflammatory responses and nuclear-transcribed mRNA catabolic process were clustered in two gene modules in AAA. The hub genes of the two modules were IL6, RPL21, and RPL7A. The expression levels of IL6 correlated positively with RPL7A and negatively with RPL21. The expression of RPL21 and RPL7A was downregulated, whereas that of IL6 was upregulated in AAA.

**Conclusions:** The expression of RPL21 or RPL7A combined with IL6 has a diagnostic value for AAA. The novel DEGs and pathways identified herein might provide new insights into the underlying molecular mechanisms of AAA.

## Introduction

Abdominal aortic aneurysm (AAA) is a degenerative vascular disease characterized by limited dilation of the aortic wall (1). The incidence of AAA has significantly increased with the increase in global aging. Aneurysm rupture leads to sudden death with an incidence of 60–85%, which is a serious threat to the health of middle-aged and elderly people (2, 3). In-depth study of the pathogenesis of AAA and looking for new biomarkers will help to provide a more rapid and convenient examination method for the diagnosis of AAA. Therefore, early diagnosis and drug intervention can be performed on AAA accurately and sensitively in the early stage of onset and provide a new molecular therapy strategy for the treatment of AAA and minimize the mortality rate. AAA has a certain genetic susceptibility. Ogata et al. (4) found that the risk of AAA in the first-degree relatives of AAA patient is nine times that of the general population. Larsson et al. (5) found that the relative risk of AAA for first-degree relatives with family history of AAA increased about twice. The polygenic nature of AAA suggests that there are still many unresearched genes and epigenetic factors that play a role in the initiation and progression of AAA (6). The occurrence and development of AAA comprise a complex pathophysiological process that involves numerous intricate molecular and cellular interaction networks (7). However, the specific mechanism has not yet been fully elucidated.

At present, bioinformatics analysis has become one of the important methods of medical scientific research (8). The big data is changing the traditional working mode of clinical scientific research workers. At the same time, it also enables clinical scientific researchers to improve their work efficiency and save scientific research resources. The integration analysis of the database also avoids the errors of small sample experimental research and increases the credibility of the research results. A similar bioinformatics analysis promoted our preliminary understanding and cognition of the pathogenesis of disease and also provided reliable ideas and directions for future specific clinical experimental research. It also provides a reliable way of thinking and direction for future clinical experimental research. High-throughput microarray platforms have emerged as a promising and efficient tool with which significant genetic or epigenetic alterations in disease states can be explored and promising biomarkers for the diagnosis and prognosis of diseases can be identified (9), although, there have been relevant studies that have explored the clinical value of some AAA biomarkers (10) [matrix metalloproteinase-9 (MMP-9), interferon-γ (IFN-γ), migration inhibitory factor (MIF), immunoglobulins against Chlamydia pneumoniae (IGA-CP), etc.,]. Lindholt et al. (11) suggested that IgA–CP and macrophage MIF can be new markers of aneurysmal progression involved in the aortic wall degradation in AAA. Pan et al. (12) suggested that MIF may be potentially involved in proinflammatory cytokine in the pathogenesis of AAA. Liu et al. (13) found that embelin can inhibit AAA through decreasing IL-6-induced STAT3 and NF-κB inactivation. Nishihara et al. (14) found that the level of IL6 in human AAA tissue may reflect the degree of ongoing inflammatory cell flux. However, Karlsson et al. (15) suggested that there was no correlation found between levels of circulating IL6, MMP-9, and C-reactive protein (CRP) and the expansion of small-diameter AAAs, indicating no clinical use of these markers in AAA surveillance. Since AAA is a complex disease, an integrated view of biomarker development is essential. In addition, the search for such a biomarker may lead to fresh insight into the pathophysiological mechanisms of AAA and may influence the future proteomic studies of tissue, serum, and plasma from affected patients. This study aimed to clarify the key molecules and pathways involved in the pathogenesis of AAA through bioinformatics, to determine the pathogenesis of AAA, and to discover key molecules with clinical value.

## Materials and Methods

### Data Collection and Analysis of Differentially Expressed Genes

The mRNA expression profiling datasets GSE47472 and GSE57691 both based on GPL10558 (Illumina HumanHT-12 V4.0 expression bead chip) were downloaded from the GEO database (https://www.ncbi.nlm.nih.gov/geo/). GSE47472 and GSE57691 are uploaded successively by the same team, and both are detected by the same platform. This ensures not only the consistency of the sample source standard but also the consistency of the sequencing process. The GSE47472 dataset contained 14 abdominal aneurysm samples and eight normal aortic samples. The GSE57691 dataset contained 49 abdominal aneurysm samples and 10 normal aortic samples. All samples are taken from the full-thickness aortic wall, which can better avoid the influence of different sampling sites on the data analysis results. The GSE47472 and GSE57691 datasets were merged and normalized using the “sva” R package. We identified differentially expressed genes (DEGs) using the “limma” package in R. Values with *p* < 0.05 and |log2Fold change (logFC)| >0.8 were considered statistically significant. The sources of the clinical samples selected in our study are relatively consistent, and they are all sequenced by the same platform. In addition, before the DEG analysis, we used the “SVA” R package to normalize the two datasets. Different from the previous study (16), we chose GSE47472 and GSE57691 datasets for integration and further bioinformatics analysis, used |log2Fold change (logFC)| >0.8 as another threshold, and used the adjusted *p*-value.

### Functional and Pathway Enrichment of Gene Modules

We explored the biofunctions of the DEGs Gene Ontology [GO; we can get what our target gene is mainly related to the three levels of cellular component (CC), molecular function (MF), and biological process (BP)] enrichment and Kyoto Encyclopedia of Genes and Genomes (KEGG; we can know which signal pathways of the target gene are involved) pathways using the “Bioconductor” and “Cluster Profiler” package in R 3.6.1 software (17). KEGG annotations included 29.0% (9/31) of upregulated genes and 10.1% (9/89) of downregulated genes. Functional pathway analysis mapped genes to KEGG pathways. The Benjamini-adjusted *p* = 0.05 was set as the cutoff for screening out significant GO terms and the KEGG pathways. The protein–protein interaction (PPI) network was generated by the Cytoscape software (http://cytoscape.org/). A node in the PPI network denotes protein, and the edge denotes the interactions.

### Identification of Hub Genes in Functional Modules and Crucial Gene Mining

We used STRING database (Protein–Protein Interaction Networks Functional Enrichment Analysis; http://string-db.org) to search for the PPI pairs of DEGs in the AAA and control groups. A PPI network map was constructed using Cytoscape software. Meanwhile, molecular complex detection (MCODE) identified the most important module of the network map. The criteria for analysis were a degree cutoff of 2, MCODE scores >5, max depth of 100, k-score of 2, and node score cutoff of 0.2. Hub genes were excavated by setting the degrees. We used OmicShare to analyze hub gene clustering.

### Statistical Analysis

Results are presented as mean ± standard error of the mean. Statistical significance of the difference between two groups was determined using Student's *t*-test. The expression of AAA hub genes was compared for correlation analysis using Pearson's rho (ρ) tests. The ability of hub genes to predict AAA was determined using receiver operating characteristics (ROC) curves. All data were statistically analyzed using SPSS software, version 23.0 (IBM Corp., Armonk, NY, USA). Values with *p* < 0.05 were considered significantly different.

## Results

### Identification of Differentially Expressed Genes

**Figure 1:**
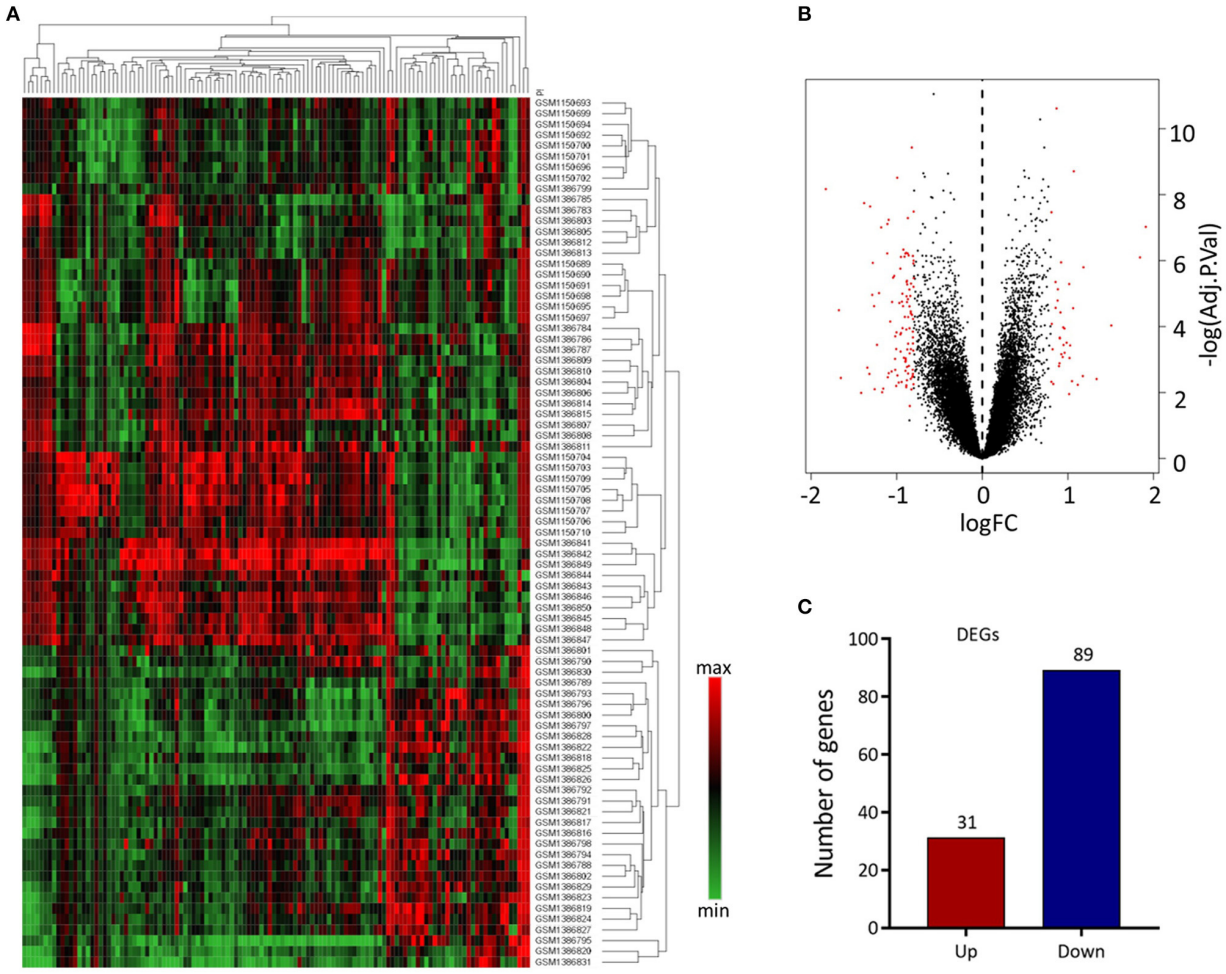
Differential expressed gene (DEG) analysis. **(A)** Heatmap of 120 DEGs. The diagram presents the result of a two-way hierarchical clustering of all the DEGs and samples. Each row in the heatmap represents a sample, and each column represents a gene. The color scale at the right of the heatmap represents the raw Z-score ranging from green (low expression) to red (high expression). **(B)** Volcano map of DEGS. Magenta dots represent genes with |logFC| > 0.8 and adj.Pval < 0.05. The red nodes on the left represent downregulated DEGs and on the right represent upregulated DEGs; the black nodes represent genes with *p*-value > 0.05. **(C)** The number of the downregulated and upregulated genes.

We analyzed 63 AAA and 18 normal samples of full-thickness aortic walls. A comparison of DEGs between the two groups using the limma package identified 120 DEGs. Heatmaps of these DEGs used in hierarchy cluster analysis suggested that they could distinguish between the AAA and normal aortae (Figure 1A). We analyzed the GSE47472 and GSE57691 datasets using R, and the differences between AAA and normal tissues were visualized in volcano plots (Figure 1B). We found 31 upregulated and 89 downregulated genes in the two gene expression groups (Figure 1C).

### Functional and Pathway Enrichment Analysis of Differentially Expressed Genes

**Figure 2:**
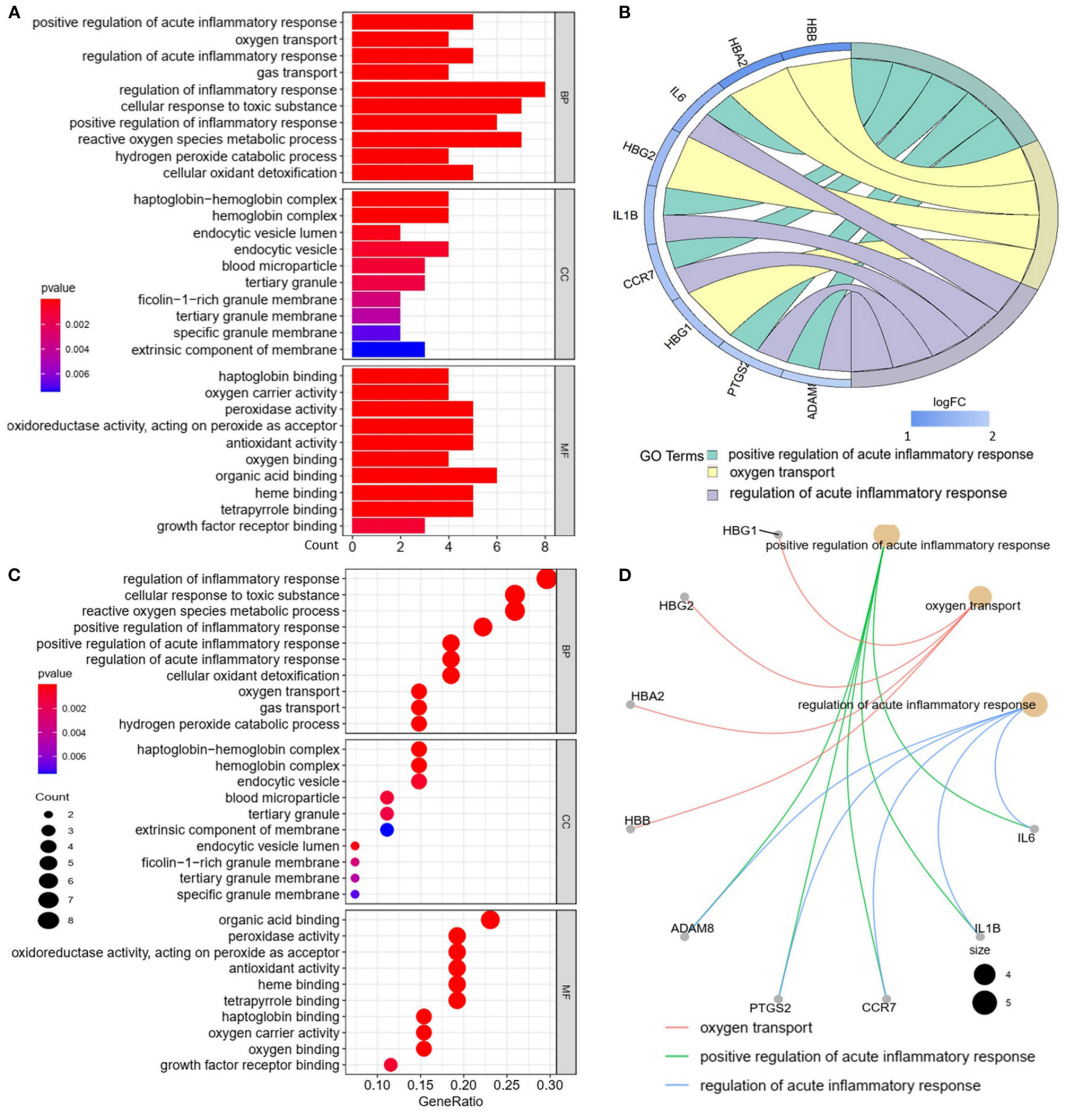
Gene Ontology (GO) analyses of the upregulated differentially expressed genes (DEGs) between abdominal aortic aneurysm (AAA) and health control (Normal) in the biological processes (BPs), cellular components (CCs), and molecular functions (MFs). **(A)** GO histogram plot: the y-axis represents the term where genes are enriched, and the x-axis represents the gene enrichment number to the corresponding term. The color of the column represents the *p*-value. **(B)** The top 3 enriched GO terms. GO chord plot: the genes are linked *via* ribbons to their assigned terms. Red coding next to the selected genes indicates logFC. **(C)** GO bubble plot: the y-axis represents the term where genes are enriched, and the x-axis represents the ratio of term genes to the total genes. The color of the dot represents the *p*-value, and the size of the dot represents the number of gene enrichment. **(D)** The top 3 enriched GO terms. GO cnetplot: the genes are linked *via* lines to their assigned terms. The size of the dot indicates the number of genes enriched to the corresponding term.

**Figure 3:**
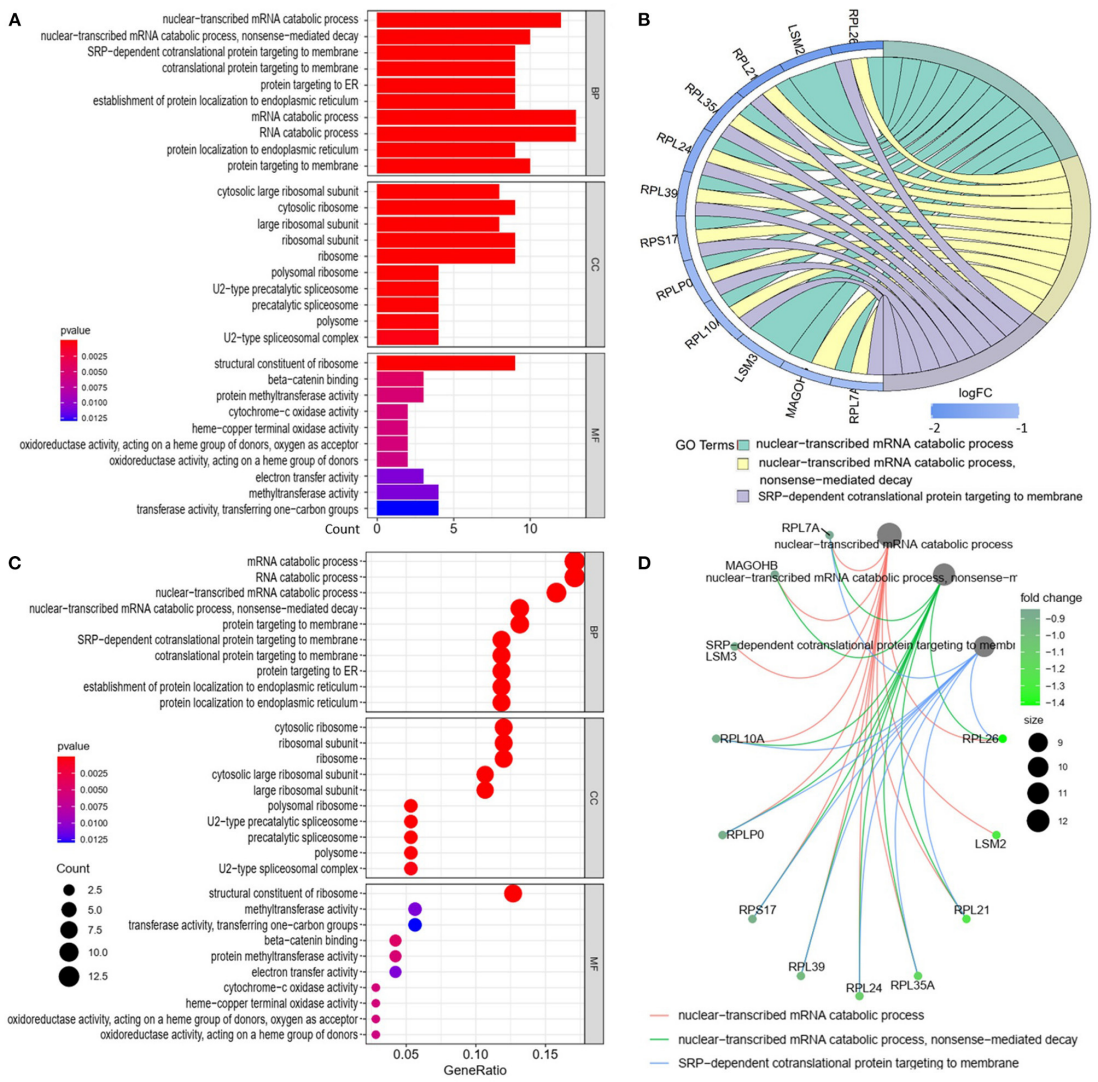
Gene Ontology analyses of the downregulated differentially expressed genes (DEGs) between abdominal aortic aneurysm (AAA) and health control (Normal) in the biological processes (BPs), cellular components (CCs), and molecular functions (MFs). **(A)** GO histogram plot: the y-axis represents the term where genes are enriched, and the x-axis represents the gene enrichment number to the corresponding term. The color of the column represents the *p*-value. **(B)** The top 3 enriched GO terms. GO chord plot: the genes are linked *via* ribbons to their assigned terms. Blue coding next to the selected genes indicates logFC. **(C)** GO bubble plot: the y-axis represents the term where genes are enriched, and the x-axis represents the ratio of term genes to the total genes. The color of the dot represents the *p*-value, and the size of the dot represents the number of gene enrichment. **(D)** The top 3 enriched GO terms. GO cnetplot the genes are linked *via* lines to their assigned terms. The size of the dot indicates the number of genes enriched to the corresponding term. The depth of the green dots indicates fold changes.

**Figure 4:**
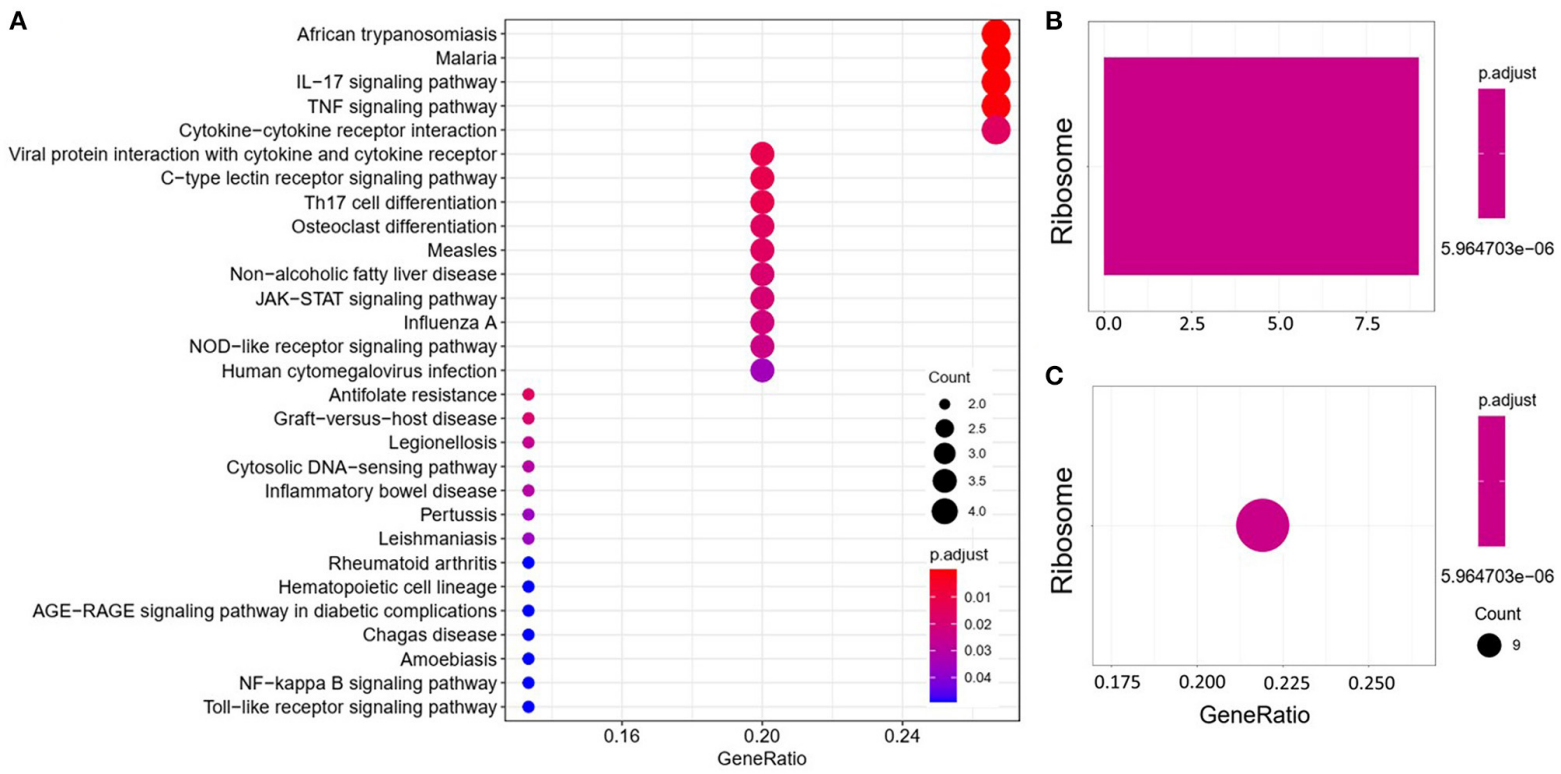
Kyoto Encyclopedia of Genes and Genomes (KEGG) analyses of the upregulated **(A)** and downregulated **(B,C)** differentially expressed genes (DEGs) between abdominal aortic aneurysm (AAA) and health control (Normal).

We analyzed the GO and KEGG pathways using the Cluster-Profiler package in R to determine the function of the 120 differentially expressed mRNA. Enrichment analyses of the 31 upregulated DEGs (Figure 2) showed that the top three terms in BPs included positive regulation of acute inflammatory response, oxygen transport, and regulation of acute inflammatory response. The CC groups were haptoglobin–hemoglobin complex, hemoglobin complex, and endocytic vesicle lumen. The MFs in AAA mainly focused on haptoglobin binding, oxygen carrier activity, and peroxidase activity (Table 1). Enrichment analyses of the 89 downregulated DEGs (Figure 3) showed that the top three terms in BPs included nuclear-transcribed mRNA catabolic process, nuclear-transcribed mRNA catabolic process, non-sense-mediated decay, and signal recognition particle (SRP)-dependent cotranslational protein targeting to membrane. The CC groups were cytosolic large ribosomal subunit, cytosolic ribosome, and large ribosomal subunit. The MFs in AAA mainly focused on the structural constituents of ribosome (Table 1). The KEGG pathway findings of the upregulated DEGs revealed that mRNA was mainly involved in the IL17 and TNF signaling pathways (Figure 4A, Table 2). The KEGG pathway findings of the downregulated DEGs revealed that the mRNA was mainly involved in the ribosome (Figures 4B,C; Table 2).

**Table 1:** The biological process, cellular component, and molecular functions in enriched analysis of differentially expressed genes between AAA and control (top 3 according to adj.P value).

	**Term**	**Name**	**Count**	**adj.Pval**
**Upregulated**
BP	GO:0002675	Positive regulation of acute inflammatory response	5	1.03^E−06^
	GO:0015671	Oxygen transport	4	2.74^E−06^
	GO:0002673	Regulation of acute inflammatory response	5	2.74^E−06^
CC	GO:0031838	Haptoglobin–hemoglobin complex	4	3.03^E−08^
	GO:0005833	Hemoglobin complex	4	3.03^E−08^
	GO:0071682	Endocytic vesicle lumen	2	4.98^E−03^
MF	GO:0031720	Haptoglobin binding	4	9.02^E−08^
	GO:0005344	Oxygen carrier activity	4	2.14^E−07^
	GO:0004601	Peroxidase activity	5	4.44^E−07^
**Downregulated**
BP	GO:0000956	Nuclear-transcribed mRNA catabolic process	12	3.59^E−08^
	GO:0000184	Nuclear-transcribed mRNA catabolic process, non-sense-mediated decay	10	3.59^E−08^
	GO:0006614	SRP-dependent cotranslational protein targeting to membrane	9	1.80^E−07^
CC	GO:0022625	Cytosolic large ribosomal subunit	8	1.60^E−08^
	GO:0022626	Cytosolic ribosome	9	3.35^E−08^
	GO:0015934	Large ribosomal subunit	8	1.04^E−06^
MF	GO:0003735	Structural constituent of ribosome	9	1.79^E−05^

*AAA, abdominal aortic aneurysm; BP, biological process; CC, cellular component; MF, molecular function; SRP, signal recognition particle*.

**Table 2:** The Kyoto Encyclopedia of Genes and Genomes (KEGG) pathway analysis of differentially expressed genes (DEGs).

	**Term**	**Count**	**adj.Pval**	**Genes**
UP	IL17 signaling pathway	4	6.53^E−04^	*IL6, FOSB, IL1B, PTGS2*
	TNF signaling pathway	4	9.78^E−04^	*IL6, IL1B, PTGS2, SOCS3*
Down	Ribosome	9	5.96^E−06^	*RPL26, RPL21, RPL24, RPL7A, RPL35A, RPL39, RPLP0, RPS17, RPL10A*

### Key Candidate Gene Identification Using Differentially Expressed Gene Protein–Protein Interaction Network

**Figure 5:**
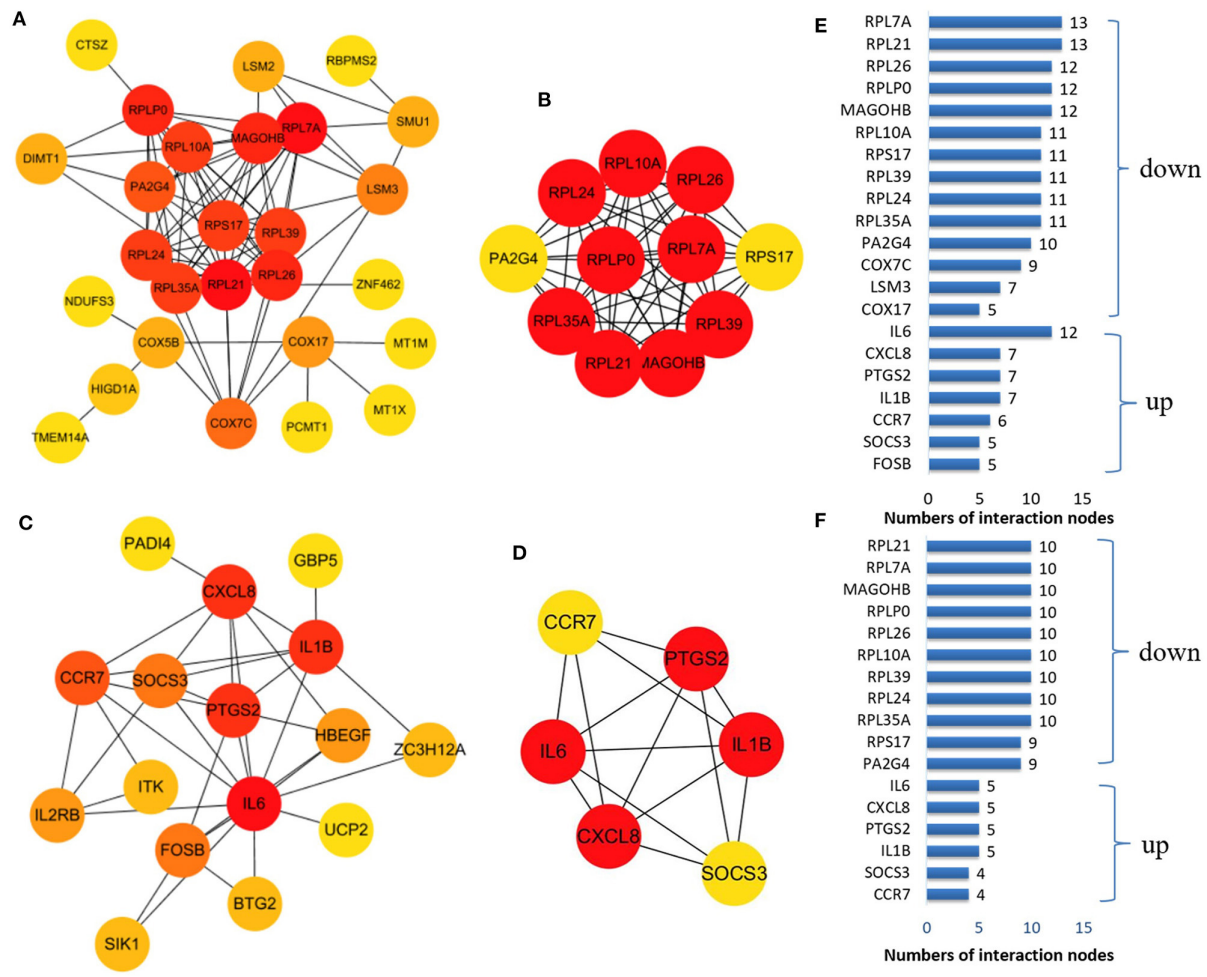
The protein–protein interaction (PPI) network constructed *via* the STRING database for the DEP with the colors of nodes representing the degree of gene interaction. PPI network of the downregulated DEGs **(A)** and the upregulated DEGs **(C)** identified from GSE47472 and GSE57691. The sub-networks were identified by Cytoscape MCODE plug-in [**(B)**, upregulated; **(D)**, downregulated]. **(E)** Genes from the upregulated and downregulated DEGs based on the PPI network analysis with the degree of interaction in the PPI network. **(F)** Genes from the two sub-networks with the degree of interaction in the PPI network.

We established a PPI network of upregulated and downregulated DEGs using STRING and visualized it using Cytoscape (Figures 5A,C). We also analyzed modules using the Cytoscape plugin and MCODE and identified the top modules with MCODE scores >5 and >5 nodes (Figures 5B,D). The significant node of the upregulated and downregulated DEGs based on PPI network analysis contained 14 and 7 nodes, respectively (Figure 5E). The significant modules of the upregulated and downregulated DEGs based on module analysis of the PPI network contained 11 and six nodes, respectively (Figure 5F). The node degrees in the upregulated and downregulated DEGs were most significant for IL6, RPL21, and RPL7A, which also were the most significant in the two modules. Functional enrichment analysis of these hub genes in the modules was mainly related to the ribosome and TNF and IL17 signaling pathways (Table 2).

### Hub Genes Have Clinical Significance for Abdominal Aortic Aneurysm

**Figure 6:**
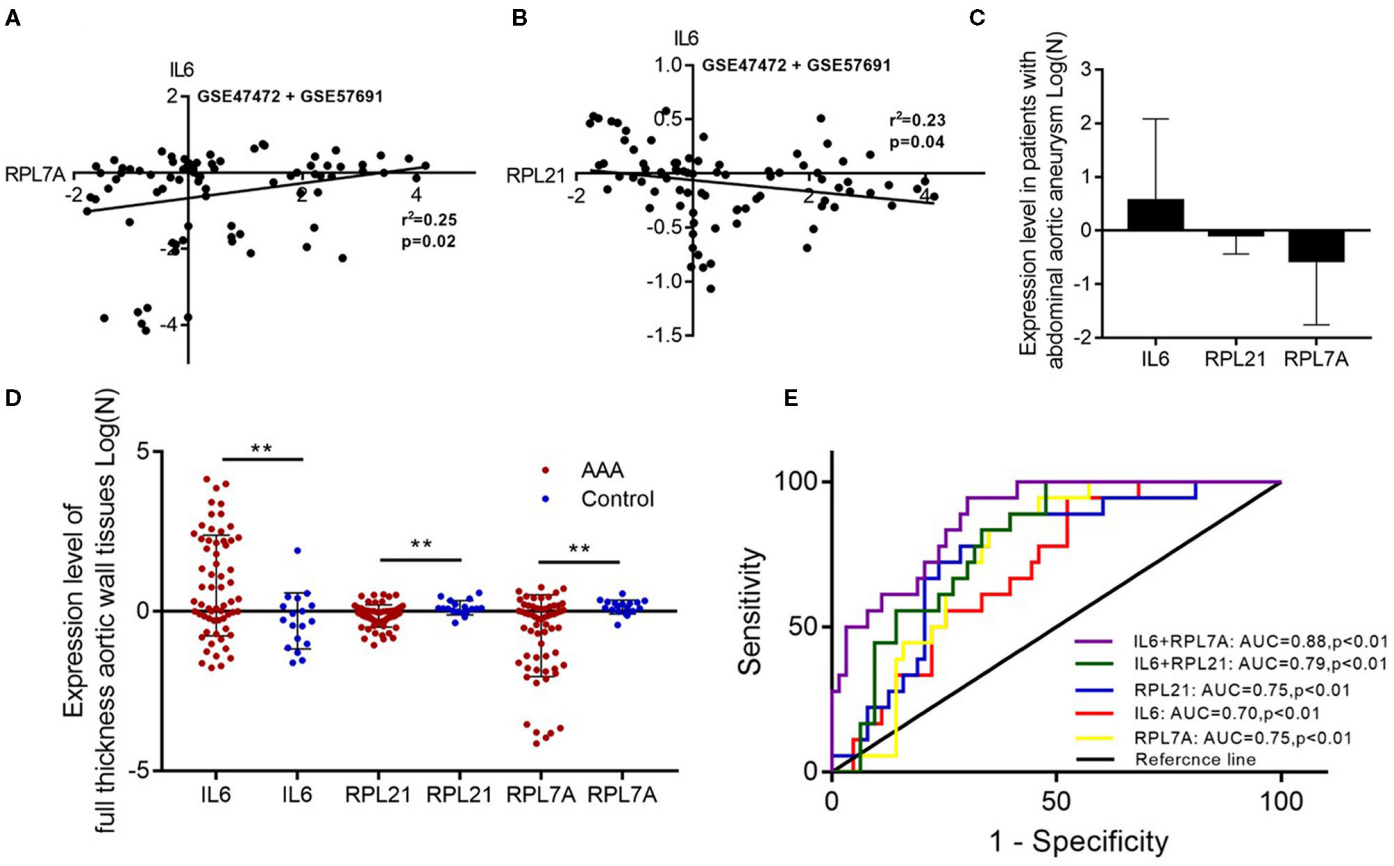
The relationship and their diagnostic value between differentially expressed genes (DEGs) in the abdominal aortic aneurysm (AAA). **(A)** The expression levels of IL6 and RPL7A showed the positive correlation in the AAA. **(B)** The expression levels of IL6 and RPL21 showed the negative correlation in the AAA. **(C)** The expression of IL6 was upregulated, and RPL21 RPL7A were downregulated in the AAA. **(D)** The expression levels of the key DEGs between the AAA and normal group in the GSE47472 and GSE57691 datasets. **P < 0.01. **(E)** Receiver operating characteristic analysis of the sensitivity and specificity of the predictive value of the combined IL6 and RPL21; IL6 and RPL7A expression model, IL6 expression model, RPL7A expression model, and RPL21 expression model.

The expression of the hub genes RPL7A and RPL21 was downregulated, whereas that of IL6 was upregulated in AAA. The expression of IL6 correlated positively with RPL7A but negatively with RPL21 (Figures 6A–D). The area under the ROC curve indicated that the three genes have a potential diagnostic value and might serve as biomarkers of AAA (Figure 6E). Their diagnostic power increased when combined with the expression levels of related molecules; however, this requires further validation.

## Discussion

This study mined, sorted, and screened chip data of human AAA genes in the Gene Expression Omnibus (GEO) database using bioinformatics, and further statistical methods were applied to extract meaningful information. We created a network map of molecular and cellular interactions. We also analyzed gene expression in samples of full-thickness aortic walls from patients without AAA (control) and with AAA to explore potential DEGs between them using bioinformatics analysis. We compared key disease-related genes between AAA and normal aortic walls in 81 results derived from two pairs of chips obtained from GEO data and then analyzed their biological relevance using gene enrichment. We found that IL6, RPL21, and RPL7A were key DEGs with pathogenic or therapeutic relevance to AAA. These three DEGs can be the key biomarkers with mechanistic relevance to AAA pathogenesis and progression.

The GO-BP terms and KEGG pathways for the upregulated DEGs were mainly associated with inflammatory responses. These results indicated that immune and inflammatory processes characterize the development of AAA, which was similar to previous findings. The occurrence, development, and prognosis of AAA are closely associated with aortic inflammation (18). Vascular damage in most cardiovascular diseases is closely related to inflammation, which can result in the necrosis of vascular endothelial cells, thus destroying the structure of blood vessel walls (19). During the occurrence and development of AAA, monocytes in the adventitia of the abdominal aorta can secrete IL6 under the action of fibroblasts to promote their own differentiation into macrophages and further promote macrophages (20). Phage cells become pathogenic macrophages. The classic inflammatory factor, IL6, mediates related signal transmission and binds to receptors on various types of cells to amplify and aggravate the degree of the inflammatory response (21, 22). Several reports stated that IL6 is a crucial gene involved in AAA development and pathogenesis. Liu et al. (13) found that embelin can inhibit AAA through decreasing IL6-induced STAT3 and NF-κB inactivation. Nishihara et al. (14) suggested that the level of IL6 in human AAA tissue may reflect the degree of ongoing inflammatory cell flux. The results of the studies of Liu et al. and Nishihara et al. were all through research of AAA mouse models. Our results suggested that IL6 expression is increased in the arterial wall tissues of patient AAA compared with control patients. IL6 was a key differential gene affecting the occurrence and development of AAA, which is consistent with the results of other studies. Therefore, IL6 can be regarded as a potentially crucial gene with a diagnostic value in the pathogenesis of AAA. Our results not only screen out the key differential genes that affect the development of AAA but also include BPs, CCs, and MFs, as well as signaling pathways and gene interaction modules. These results are helpful for our preliminary understanding of the occurrence and development mechanism of AAA and could provide reliable ideas and directions for future specific experimental research. Further, research is now needed to understand the exact role of IL6 in order to establish improved diagnostic and therapeutic strategies for AAA.

The GO-BP terms and KEGG pathways in the downregulated DEGs were mainly associated with nuclear-transcribed mRNA catabolic process and ribosomes. Accumulated evidence shows that ribosomal proteins play important roles in regulating the occurrence and development of many diseases (23). Ribosomes participate not only in protein synthesis but also in ribose functions *in vitro*, including cell growth, differentiation, and apoptosis (24). Both cell division and proliferation, and apoptosis are inseparable from the regulation of ribosomes. Mutations and deletions of ribosomal proteins can lead to abnormal cell metabolism, cell growth arrest, and even death (23). The regulation of ribosomal protein genes has widespread effects on the physiological functions of cells without affecting protein translation (25). For example, they can change the structure and function of cell membranes, regulate cell metabolism, cause abnormal cell growth, induce apoptosis, and trigger various pathophysiological processes (26). Recently, more and more studies have shown that abnormal expression of ribosomal protein can lead to functional changes of ribosomal and/or ribosomal protein and then lead to the occurrence of diseases such as tumors. The changes of some ribosomal proteins can affect the binding of the corresponding large and small ribosomal subunits with other ribosomal proteins and then endanger the survival of the whole cell. In addition to being detected in tissues, ribosomal protein can also be detected in body fluids, blood, and feces. For example, the level of RPS27L in feces is positively correlated with cancer tissue, which can be used as a potential biomarker for early diagnosis (27). The level of RPL19 in the stool combined with the level of serum carcinoembryonic antigen (CEA) can predict the prognosis of patients with colorectal cancer. Rasmussen et al. observed the differences in expression of mRNA of 18S and 28S in the blood and the brain in both healthy elderly individuals and Alzheimer's disease (AD) patients. They believe that the changes in rRNA present in AD patients are tissue-specific (28). Low expression and insufficient ribosomal proteins can lead to tumors. For example, a deficiency of any of its 11 ribosomal proteins due to low gene expression levels can lead to malignant tumors in zebrafish (29). Wang et al. found that RPL7A was one of the most significant survival-predicting differentiation-related genes [glioblastoma (GBM) cell differentiation-related genes (GDRGs)], and low expression of RPL7A was associated with poor overall survival (OS) in GBM patients (30). Bolze et al. suggested that heterozygous coding mutations in ribosomal protein SA underlie most cases of isolated congenital asplenia. Their unbiased analysis of exomes revealed heterozygous mutations in ribosomal protein SA in 18 patients from eight kindreds (31). Farrar et al. (32) suggested that ribosomal protein gene deletion should be considered a component of the initial genetic evaluation in cases of suspected diamond-blackfan anemia. A study has found that PABPC1 and RPL13A may be potential biomarkers of cervical intraepithelial neoplasia 1 (CIN1) and potential targets of treatment pathways (33). Mutations in the RPS27 promoter may be a mechanism of gene expression regulation in patients with melanoma, which may have prognostic and predictive significance (34). Our results suggested that the expression of RPL21 and RPL7A in the arterial walls of patients with AAA was decreased as compared with that of the control group, and that it can be regarded as a crucial gene in the pathogenesis of AAA having a potential diagnostic value. The RPL21 gene encodes ribosomal protein 21, which is an important part of the large 60S ribosomal subunit (35). The RPL21 genes in various organisms have been cloned and identified, which have led to the finding that RPL21 is associated with the formation and development of mouse tooth germ (36). Mutations in the RPL21 gene can cause hereditary alopecia (37). Ribosomal protein RPL7A is a component of the 60S ribosomal subunit. Its coding gene is located on chromosome 9q34, and it is closely related to cell growth and differentiation. In addition, RPL7A can specifically bind to thyroid hormone and retinoic acid receptors and inhibit their transcriptional activation (38). Thyroid hormones can play an important role in regulating cell proliferation, differentiation, and death (39). In addition, Li et al. (40) found that the increase of caspase-8 activities and the loss of mitochondrial membrane potential after RPL21 silencing indicates that the RPL21 gene may be involved in caspase-8-related mitochondrial apoptosis. Wang et al. (30) suggested that RPL7A was significantly upregulated in macrophages. Therefore, we speculate that RPL21 and RPL7A may regulate the apoptosis of smooth muscle cells by affecting the inflammatory response of vascular wall of AAA and then affect the occurrence of AAA. We believe that RPL21 and RPL7A have a certain possibility as potential targets of AAA. Of course, the specific mechanisms by which RPL21 and RPL7A may be used as AAA markers still need to be explored and confirmed by subsequent further experimental studies.

As a classic inflammatory factor, IL6 can amplify and aggravate the degree of the inflammatory response. The level of IL6 reflects the severity of the inflammatory response to a certain extent. Both RPL21 and RPL7A are important parts of the large 60S ribosomal subunit; however, their respective functions have not yet been fully elucidated. Li et al. (40) found that RPL21 gene may be involved in caspase-8-related mitochondrial apoptosis. In view of the relationship between inflammatory response and apoptosis, we hypothesized that RPL21 might be involved in reducing inflammatory response. Wang et al. suggested that RPL7A was significantly upregulated in GBM cells and macrophages (30). Given the effect of chronic inflammatory response on tumors, we hypothesized that RPL7A might be associated with aggravating inflammatory response. Therefore, we hypothesized that there may be different effects of RPL21 and RPL7A on the inflammatory response. This is also consistent with our results. In our study, the expression of IL6 correlated positively with RPL7A but negatively with RPL21. So we speculate that although RPL21 and RPL7A are both downregulated in AAA, their effects on inflammatory response may be different, or even their effects on inflammatory response may be completely different. This needs to be discussed and verified by further research in the later stage. Our results suggest that the expression of RPL21 or RPL7A combined with IL6 has a diagnostic value for AAA. In addition, our results also involve the corresponding signal pathways and cellular functions, which will be helpful for further exploration of the AAA mechanism and the new research ideas. In general, although, the results in our study are based on chip data, we have applied different statistical methods to verify our results within the data and show our new findings. These results, to some extent, could be enlightening for the subsequent mechanism studies. Of course, the specificity of ribosomal protein and IL6 for AAA biomarkers needs further verification and discussion in subsequent studies.

This study had several limitations. The key differential genes and their pathways we have identified have not been confirmed by *in vitro* studies or other functional studies; however, this will be an area for further research. In addition, the patient's clinical medication information data were not provided by the uploader, so the effect of drug on extracellular matrix (ECM) composition could not be considered in this study. Furthermore, although, the results in our study were based on chip data, we have applied different statistical methods to verify our results within the data and show our new findings. These results, to some extent, could be enlightening for the subsequent mechanism studies.

We sequenced the GEO datasets GSE47472 and GSE57691 based on GPL10558 (Illumina HumanHT-12 V4.0 expression bead chip) from full-thickness aortic wall tissues with high consistency and reliability. We then merged and normalized the datasets, constructed a co-expression network, detected gene modules, and identified three hub genes (IL6, RPL21, and RPL7A) and three signal pathways (ribosome, IL17, and TNF signaling) that differed between AAA and normal aortae. These hub genes and signaling pathways might have crucial biological functions in the pathogenesis of AAA. We believe that this finding has enlightening significance for further mechanism research. Further, studies are needed to elucidate their potential role as diagnostic, prognostic, or therapeutic biomarkers in AAA.

## Data Availability Statement

The datasets presented in this study can be found in online repositories. The names of the repository/repositories and accession number(s) can be found in the article/Supplementary Material.

## Author Contributions

EW did the literature research and wrote a part of the manuscript. DX wrote a part of the manuscript. YZ designed the tables. XS drew the figures. XS contributed to the idea of the manuscript and wrote the first draft of the manuscript. LW provided critical feedback. WF and DG guided the writing. All the authors reviewed the manuscript and approved the submitted version.

## Conflict of Interest

The authors declare that the research was conducted in the absence of any commercial or financial relationships that could be construed as a potential conflict of interest.

## Supplementary Material

Supplementary Figure 1 Normalize the merged GSE47472 and GSE57691 chip data in the GEO database.

Supplementary Table 1 The value of differential gene expression analysis results.

Supplementary Table 2 The most significant modules from the PPI network in the up and down regulation DEGs.
