# Supplementary figures and images for: Bioinformatics Analysis Reveals the Potential Diagnostic Biomarkers for Abdominal Aortic Aneurysm
Source: Front Cardiovasc Med. 2021 Jul 20;8:656263. doi: 10.3389/fcvm.2021.656263 (PMC8329524; doi:10.3389/fcvm.2021.656263)

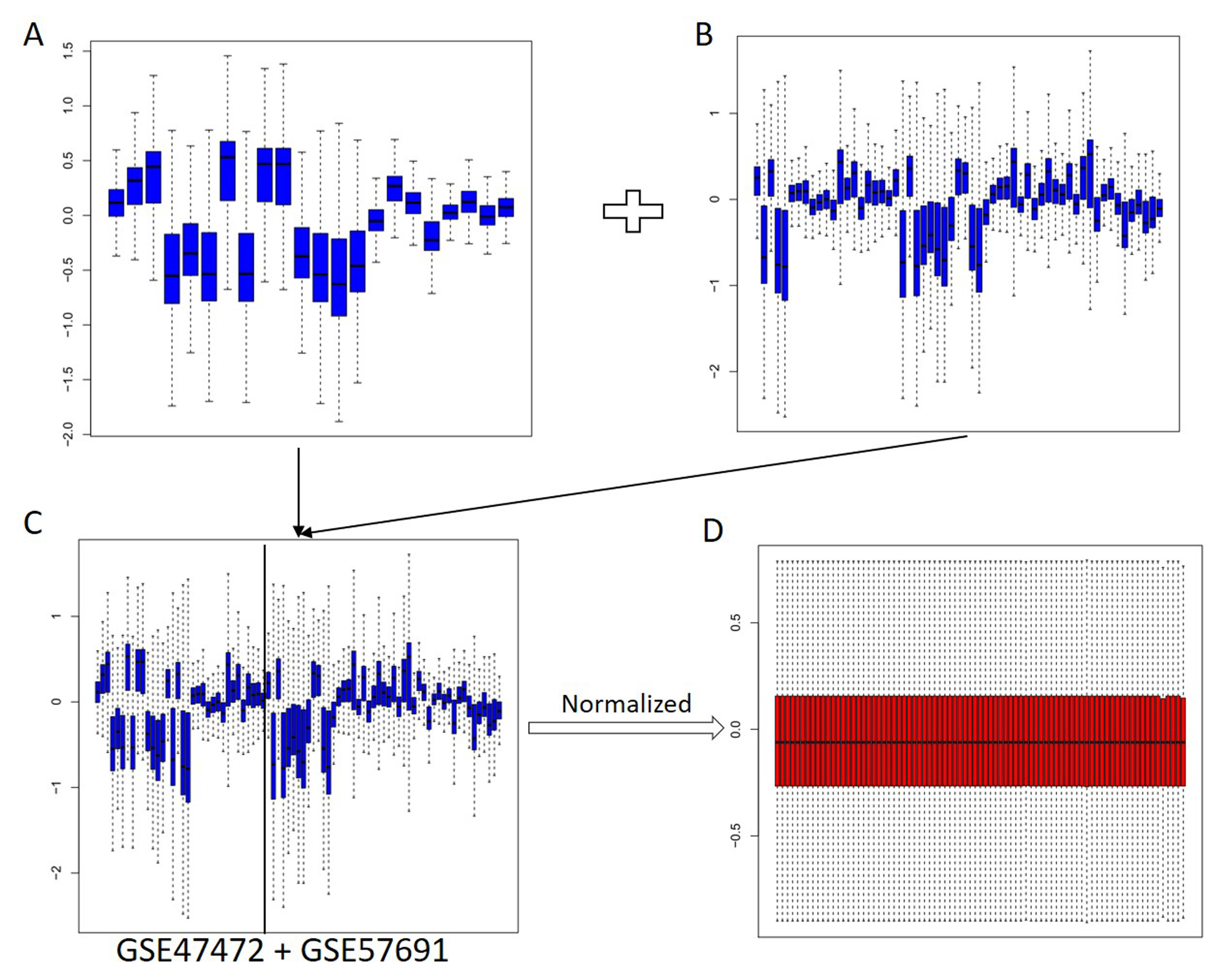

Supplement: Supplementary Figure 1 — Normalize the merged GSE47472 and GSE57691 chip data in the GEO database. [file Image_1.TIF]
