# Supplementary material for: Bioinformatics Analysis Reveals the Potential Diagnostic Biomarkers for Abdominal Aortic Aneurysm
Source: Front Cardiovasc Med. 2021 Jul 20;8:656263. doi: 10.3389/fcvm.2021.656263 (PMC8329524; doi:10.3389/fcvm.2021.656263)
Supplement: Supplementary Table 1 — The value of differential gene expression analysis results. [file Data_Sheet_1.docx]

**Supplementary Table 1: The value of differential gene expression analysis results**

| **UP-regulation** | | | | | **Down-regulation** | | | | |
| --- | --- | --- | --- | --- | --- | --- | --- | --- | --- |
| **Gene** | **logFC** | **AveExpr** | **t** | **adj.P.Val** | **Gene** | **logFC** | **AveExpr** | **t** | **adj.P.Val** |
| HBB | -1.90954 | 0.024761 | -7.23724 | 9.47E-08 | DIMT1L | 0.800496 | -0.06923 | 6.153075 | 3.4E-06 |
| HBA2 | -1.84216 | 0.100316 | -6.61508 | 7.93E-07 | ITGB1BP1 | 0.801411 | -0.04528 | 7.566282 | 3.29E-08 |
| SIK1 | -1.507 | 0.434958 | -5.06962 | 9.31E-05 | BRP44 | 0.802202 | -0.03506 | 6.556038 | 9.98E-07 |
| IL6 | -1.33497 | 0.503509 | -3.67972 | 0.003901 | PCMT1 | 0.803359 | -0.0874 | 5.959345 | 6.26E-06 |
| ARL17P1 | -1.18154 | 0.074812 | -6.39081 | 1.59E-06 | DACT3 | 0.803751 | -0.24609 | 3.521355 | 0.005651 |
| HBG2 | -1.17408 | 0.517097 | -3.76253 | 0.003179 | SMYD4 | 0.80468 | -0.01668 | 6.476191 | 1.21E-06 |
| FOSB | -1.11486 | -0.05491 | -3.50714 | 0.005846 | TMED4 | 0.806142 | -0.29606 | 4.453879 | 0.000538 |
| IL8 | -1.04768 | 0.329106 | -3.61658 | 0.004536 | SYNC1 | 0.808784 | -0.00375 | 5.219173 | 5.92E-05 |
| IL1B | -1.02601 | 0.338578 | -4.5869 | 0.000374 | SMU1 | 0.810289 | -0.09649 | 4.639053 | 0.000327 |
| CCR7 | -1.01839 | 0.378427 | -4.2628 | 0.000901 | FLJ21986 | 0.810847 | -0.45645 | 3.827311 | 0.002721 |
| FAM153B | -1.01492 | -0.06996 | -6.01635 | 5.12E-06 | RBM23 | 0.81108 | -0.05294 | 6.665165 | 7.16E-07 |
| HBG1 | -1.01432 | 0.526441 | -3.22246 | 0.01115 | PALLD | 0.811834 | -0.39041 | 3.941019 | 0.002062 |
| FCN1 | -0.96094 | 0.057121 | -4.39971 | 0.000626 | BRCC3 | 0.81323 | -0.12009 | 3.769238 | 0.003144 |
| PADI4 | -0.95952 | 0.27622 | -4.99794 | 0.000115 | CBX6 | 0.821457 | -0.16211 | 5.314537 | 4.45E-05 |
| SOCS3 | -0.94855 | 0.241006 | -4.5988 | 0.000363 | HES6 | 0.823474 | 0.00231 | 8.989449 | 3.71E-10 |
| G0S2 | -0.94494 | 0.222244 | -5.02595 | 0.000106 | KIAA0367 | 0.82838 | -0.28218 | 3.36668 | 0.008068 |
| SLC2A3 | -0.93899 | 0.309343 | -4.29095 | 0.000841 | UNC84B | 0.833123 | -0.18146 | 6.025565 | 4.97E-06 |
| UCP2 | -0.92309 | 0.211912 | -4.3647 | 0.000692 | NPTX2 | 0.834571 | -0.03661 | 4.489671 | 0.000487 |
| C2orf19 | -0.92001 | -0.16815 | -6.50061 | 1.12E-06 | TMEM14A | 0.834696 | 0.055915 | 5.346016 | 4.01E-05 |
| ZC3H12A | -0.91284 | 0.220076 | -5.36865 | 3.73E-05 | CD63 | 0.835599 | -0.4032 | 3.684553 | 0.003857 |
| BTG2 | -0.90547 | 0.211125 | -4.75811 | 0.00023 | ZNF575 | 0.841088 | -0.06212 | 5.752052 | 1.18E-05 |
| ITK | -0.90481 | 0.324628 | -4.77868 | 0.000217 | RPL7A | 0.847143 | -0.5682 | 2.836765 | 0.025644 |
| HBEGF | -0.90174 | 0.16061 | -4.12441 | 0.001304 | COX7C | 0.849042 | -0.34547 | 4.38456 | 0.000654 |
| PTGS2 | -0.89587 | 0.102794 | -4.04506 | 0.001585 | PA2G4 | 0.850562 | -0.00909 | 5.004753 | 0.000113 |
| IL2RB | -0.87935 | 0.113204 | -5.65259 | 1.63E-05 | MAGOHB | 0.85098 | 0.040773 | 5.510444 | 2.44E-05 |
| ANGPTL6 | -0.8674 | -0.01502 | -9.73964 | 2.43E-11 | HIGD1A | 0.851621 | -0.07892 | 5.897139 | 7.59E-06 |
| RPPH1 | -0.85595 | 0.030645 | -5.47716 | 2.71E-05 | SERPINA3 | 0.857694 | -0.32185 | 4.049748 | 0.001568 |
| GBP5 | -0.85518 | 0.025332 | -6.06301 | 4.43E-06 | GGNBP2 | 0.860087 | -0.05383 | 6.685985 | 6.89E-07 |
| HES3 | -0.82046 | -0.13733 | -4.4874 | 0.00049 | XPA | 0.866411 | -0.12624 | 5.262187 | 5.15E-05 |
| KIFC2 | -0.81136 | 0.070058 | -5.11075 | 8.32E-05 | TMEM181 | 0.86696 | -0.16078 | 4.631991 | 0.000332 |
| ADAM8 | -0.80685 | 0.216046 | -3.59699 | 0.004748 | MAOA | 0.867217 | -0.17208 | 3.83485 | 0.002666 |
|  |  |  |  |  | NDUFS3 | 0.869531 | -0.07954 | 7.428476 | 5.14E-08 |
|  |  |  |  |  | DUSP3 | 0.869596 | -0.231 | 5.678839 | 1.48E-05 |
|  |  |  |  |  | CDC2L6 | 0.871381 | -0.02873 | 6.733045 | 6E-07 |
|  |  |  |  |  | RBPMS2 | 0.876332 | -0.02075 | 4.481516 | 0.000497 |
|  |  |  |  |  | LSM3 | 0.877222 | -0.16714 | 6.007834 | 5.27E-06 |
|  |  |  |  |  | PGRMC1 | 0.879744 | -0.20396 | 5.612011 | 1.81E-05 |
|  |  |  |  |  | C6orf117 | 0.880304 | -0.17416 | 4.02313 | 0.00168 |
|  |  |  |  |  | BBX | 0.880451 | -0.15165 | 6.08774 | 4.1E-06 |
|  |  |  |  |  | RPESP | 0.881077 | -0.30915 | 3.644086 | 0.004259 |
|  |  |  |  |  | FHOD1 | 0.8851 | 0.096472 | 5.74462 | 1.21E-05 |
|  |  |  |  |  | ADH1A | 0.887337 | -0.35401 | 3.489031 | 0.006081 |
|  |  |  |  |  | NECAB3 | 0.887645 | -0.27683 | 4.977666 | 0.000123 |
|  |  |  |  |  | C5orf46 | 0.888857 | 0.022229 | 3.433375 | 0.006915 |
|  |  |  |  |  | GFOD1 | 0.889739 | -0.1559 | 6.750218 | 5.74E-07 |
|  |  |  |  |  | RPL10A | 0.894647 | -0.23916 | 4.213734 | 0.001039 |
|  |  |  |  |  | CAP2 | 0.895243 | -0.22738 | 4.25686 | 0.000917 |
|  |  |  |  |  | TIMP4 | 0.895442 | 0.082963 | 4.167975 | 0.001167 |
|  |  |  |  |  | RPLP0 | 0.897539 | -0.09567 | 3.913146 | 0.002203 |
|  |  |  |  |  | TRPC1 | 0.898368 | -0.08917 | 6.721789 | 6.08E-07 |
|  |  |  |  |  | RPS17 | 0.899772 | -0.45941 | 4.171827 | 0.001157 |
|  |  |  |  |  | FKBP5 | 0.90477 | -0.0371 | 5.267042 | 5.1E-05 |
|  |  |  |  |  | FOXO1 | 0.908789 | -0.03259 | 6.647652 | 7.56E-07 |
|  |  |  |  |  | COX17 | 0.916418 | -0.41052 | 4.269168 | 0.000888 |
|  |  |  |  |  | CLN5 | 0.922227 | -0.03428 | 6.818121 | 4.59E-07 |
|  |  |  |  |  | MCAM | 0.925974 | -0.02547 | 5.515391 | 2.4E-05 |
|  |  |  |  |  | C1orf19 | 0.933693 | -0.0394 | 5.80342 | 1.01E-05 |
|  |  |  |  |  | KLF9 | 0.950201 | -0.11711 | 6.634771 | 7.68E-07 |
|  |  |  |  |  | SNHG5 | 0.950412 | -0.09341 | 4.898474 | 0.000157 |
|  |  |  |  |  | LIMCH1 | 0.954621 | -0.28513 | 4.428254 | 0.000577 |
|  |  |  |  |  | KAAG1 | 0.957432 | -0.16633 | 6.364536 | 1.74E-06 |
|  |  |  |  |  | JAG1 | 0.96858 | -0.19562 | 5.592083 | 1.92E-05 |
|  |  |  |  |  | RPL39 | 0.976771 | -0.59206 | 3.575278 | 0.005003 |
|  |  |  |  |  | CSNK2A2 | 0.993657 | -0.06922 | 8.306292 | 3.1E-09 |
|  |  |  |  |  | NFIA | 1.002179 | -0.18221 | 4.908191 | 0.000152 |
|  |  |  |  |  | NOV | 1.005607 | -0.42724 | 3.470714 | 0.006332 |
|  |  |  |  |  | GMDS | 1.017047 | -0.2699 | 4.815871 | 0.000198 |
|  |  |  |  |  | RCAN2 | 1.019238 | -0.37379 | 4.2412 | 0.000962 |
|  |  |  |  |  | ITGA10 | 1.029249 | -0.05615 | 6.212357 | 2.88E-06 |
|  |  |  |  |  | CXXC5 | 1.041813 | -0.04142 | 6.163593 | 3.27E-06 |
|  |  |  |  |  | PPP1R3C | 1.059406 | -0.25043 | 3.890532 | 0.002312 |
|  |  |  |  |  | COX5B | 1.075738 | -0.40195 | 5.164994 | 7.05E-05 |
|  |  |  |  |  | RPL24 | 1.092995 | -0.60154 | 3.84439 | 0.002598 |
|  |  |  |  |  | PPP1R12C | 1.094134 | 0.041113 | 7.385386 | 5.81E-08 |
|  |  |  |  |  | SCRG1 | 1.104123 | -0.20402 | 3.729488 | 0.003454 |
|  |  |  |  |  | NR2F6 | 1.108371 | -0.07324 | 7.293123 | 7.78E-08 |
|  |  |  |  |  | C8orf59 | 1.110687 | -0.21319 | 6.726148 | 6.04E-07 |
|  |  |  |  |  | RPL35A | 1.157142 | -0.61889 | 3.388851 | 0.007652 |
|  |  |  |  |  | S100A4 | 1.17003 | -0.70254 | 3.282954 | 0.009791 |
|  |  |  |  |  | MSTN | 1.181362 | 0.113899 | 7.223867 | 9.83E-08 |
|  |  |  |  |  | MT1X | 1.230268 | -0.36369 | 4.602172 | 0.000361 |
|  |  |  |  |  | RPL21 | 1.2577 | -0.62092 | 3.372343 | 0.007957 |
|  |  |  |  |  | LSM2 | 1.279327 | -0.05081 | 6.489618 | 1.16E-06 |
|  |  |  |  |  | MT1M | 1.285971 | -0.01404 | 5.783885 | 1.09E-05 |
|  |  |  |  |  | CARM1 | 1.310428 | 0.060881 | 7.659566 | 2.28E-08 |
|  |  |  |  |  | ZNF462 | 1.380242 | -0.11414 | 7.716801 | 1.81E-08 |
|  |  |  |  |  | RPL26 | 1.415356 | -0.78856 | 3.260147 | 0.010293 |
|  |  |  |  |  | CTSZ | 1.67549 | -0.43658 | 5.424122 | 3.2E-05 |
|  |  |  |  |  | WDR82 | 1.828328 | -0.01072 | 8.060832 | 6.79E-09 |

**Supplementary Table 2: The most significant modules from the PPI network in the up and down regulation DEGs**

| **Cluster** | **Score** | **Nodes** | **Edges** | **Most degree** | **Node IDs** |
| --- | --- | --- | --- | --- | --- |
| UP | 5.6 | 6 | 14 | 5 | CCR7, SOCS3, IL1B, PTGS2 CXCL8, **IL6** |
| Down | 10.8 | 11 | 54 | 10 | RPL35A, PA2G4, RPL24, RPL39 RPL10A, RPS17, **RPL21**, RPLP0 **RPL7A**, MAGOHB, RPL26 |

Genes marked in bold indicate the most degree genes in the module.
